# Honey for acute cough in children — a systematic review

**DOI:** 10.1007/s00431-023-05066-1

**Published:** 2023-06-25

**Authors:** Ilari Kuitunen, Marjo Renko

**Affiliations:** 1 Institute of Clinical Medicine and Department of Pediatrics, University of Eastern Finland, Yliopistonranta 2, 70211 Kuopio, Finland; 2 Department of Pediatrics, Mikkeli Central Hospital, Mikkeli, Finland; 3 Department of Pediatrics, Kuopio University Hospital, Kuopio, Finland

**Keywords:** Honey, Cough, Intervention, Cough medication

## Abstract

To analyze the efficacy of using honey to treat acute cough in children. Systematic review, synthesis without meta-analysis. We searched PubMed, Scopus, CENTRAL, CINAHL, and Web of Science databases on August 15, 2022, for words honey and cough. Randomized controlled trials conducted in children were included. Risk-of-bias and evidence quality were assessed. Studies were not pooled due to lack of key information. Instead, we provided the range of observed effects for the main outcomes. Three hundred ninety-six papers were screened, and 10 studies were included. Two studies had high risk-of-bias and six had some concerns. Honey seemed to decrease cough frequency more than placebo/no treatment (range of observed effect 0.0–1.1 points) and cough medication (0.2–0.9 points). Sleep improved more often in the honey group (range of effect was 0.0–1.1) compared to placebo/no treatment and (− 0.2–1.1 points) compared to cough medication. Quality of the evidence was low to very low.

*Conclusion*: We found low quality evidence that honey may be more effective than cough medication or placebo/no treatment in relieving symptoms and improving sleep in children with acute cough. Better quality randomized, placebo-controlled blinded trials are needed to confirm the effectiveness of honey in treating acute cough in children.

*Trial registration*: CRD42022369577.

**Table:** 

**What is Known:** *• Honey has been suggested to be effective as a symptomatic treatment in acute cough.* *• Prior randomized trials have had conflicting results and thus an overview of the literature was warranted.*	
**What is New:** *• Based on low quality evidence honey may be more effective than placebo or over-the-counter medications for acute symptom reliwef in cough.* *• Future studies with better reporting are needed to confirm the results.*

**Supplementary Information:**

The online version contains supplementary material available at 10.1007/s00431-023-05066-1.

## Introduction

Cough is among the most common symptoms in children and the majority of children have at least one episode of acute cough annually [1, 2]. Acute cough is typically classified as cough lasting less than 4 weeks [3]. Acute respiratory tract infection is the most common cause of cough in children [2]. Cough affects the quality of life of both the child and his/her parents; thus, effective treatments are needed [4]. Cough medicines and syrups have not been effective in children and have also been associated with severe harm [5]. Therefore, nowadays the use of cough medication is not recommended and many guidelines prohibit the use of such products in children [6, 7].

The World Health Organization and many treatment guidelines have proposed or endorsed the use of honey to treat acute cough [8–10]. Honey has been examined in several randomized controlled trials, but the results varied. The latest systematic reviews published in 2018 and 2021 stated that honey is an effective treatment for cough and causes no severe harm; therefore, it could be used to treat acute cough [11, 12]. The latest systematic review in 2021 included both adults and children and concluded that honey is an effective alternative to antibiotics in the treatment of acute cough, even though honey’s efficacy was not compared to antibiotics [12]. Furthermore, several novel studies on honey and acute cough have been conducted after the 2018 Cochrane review, which focused on children. Therefore, we decided to update the evidence regarding the role of honey in the symptomatic treatment of acute cough in children.

## Methods

### Search strategy

PubMed (MEDLINE), Scopus, CINAHL, Cochrane Central Register of Controlled Trials (CENTRAL), and Web of Science databases were searched on August 15, 2022. The following keywords were used: honey and cough. We only included studies published in English. We did not use any filters in the search. The search results were uploaded to Covidence software (Covidence, Melbourne, Australia) for the screening process.

### Inclusion and exclusion criteria

Only randomized controlled trials were included regardless of blinding. The study intervention had to be honey or a combination product that included honey. The comparator intervention could be placebo, no treatment, or cough medication. We included studies conducted in children ranging in age between 1 and 18 years. Animal studies were excluded. Observational studies and all other studies not presenting original data were excluded.

### Review process

Two authors (IK and MR) individually screened the abstracts and full texts. Conflicts were resolved by mutual decision. Outcome data were then extracted into an Excel spreadsheet. We used the Cochrane risk-of-bias 2.0 tool to assess the risk-of-bias in the included studies [13]. Risk-of-bias figures were generated with the robvis package in R version 4.0.3. Evidence quality was assessed using the Grading of Recommendations Assessment, Development, and Evaluation, (GRADE) methodology [14].

### Outcome measures

Our main outcomes of interest were change in the cough frequency, cough severity, and children’s sleep quality after onset of the medication. Our secondary outcome was the rate of adverse events.

### Statistics

Data analyses were performed according to the Cochrane Handbook of Systematic Review Guidelines, and Review Manager software version 5.4 was used for the analyses. Originally, our plan was to either calculate the mean differences or the standardized mean differences for the main outcomes and present forest plots and funnel plots. We hypothesized that the included studies would have different dosing in the interventions and heterogenous comparator groups; thus, we decided to use a random-effects model. However, during the data extraction process, we noticed that the reporting of the main outcomes was limited, and typically standard deviations (SDs) were missing usually both for the change from baseline values and the post-intervention values. We decided not to input data using SDs from similar studies, although it would be allowed by the Cochrane handbook. As the SDs were missing in most of the studies and outcomes, most of the results would have been based on input values instead of true observed values. Thus, we utilized alternative methods to produce a systematic review of evidence without a meta-analysis and we decided to present the range of the observed effects for each outcome in tables. Thus, we presented the difference between the intervention and the comparator group as a change from baseline and the likely overall direction of this difference. Due to the lack of SD reporting in the original studies, we were unable to calculate any uncertainty estimates.

We reported our systematic review and meta-analysis findings according to the Preferred Reporting Items for Systematic Reviews and Meta-Analyses (PRISMA) and we provided the PRISMA statement in a supplementary file (Supplement 1) [15]. Because we did not perform a quantitative analysis, we also utilized the Synthesis without meta-analysis (SWiM) guideline [16].

### Protocol registration

We registered our protocol in Prospero, registration number: CRD42022369577.

## Results

**Fig. 1:**
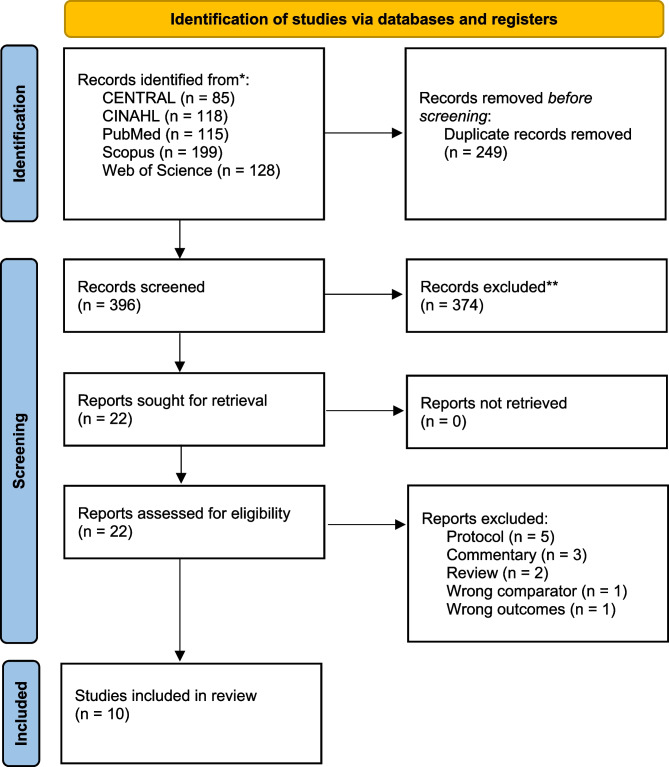
Flow chart of the study selection process

Our search retrieved 396 results; after screening the titles and abstracts, 22 studies were further assessed. Ultimately, 10 of those studies were included in this review [17–26], as seen in Fig. 1. Of the 10 included studies, five were double-blinded, two were single-blinded, and three were unblinded (Supplementary Table 1). Studies were conducted on every continent except South America. The inclusion and exclusion criteria were relatively similar in all the studies. Pure honey was the intervention in seven of the studies and three studies used products that contained honey. Funding information was reported by seven of the studies; of these studies, three reported no specific funding. One of the included studies was funded by a national honey association. The authors did not report potential conflicts of interests in two studies. Two studies reported that the authors had a relationship with companies that provided the study medication (Supplementary Table 1).

The number of participants in the included studies ranged from 68 to 270 (Table 2). The mean age of the children ranged from 2.4 to 5.4 years. Overall, the cough severity scores at baseline were comparable between the intervention and study groups (Table 1).

**Table 1:** Baseline characteristics of the children in the intervention and comparator groups of the included studies at the time of the randomization

	Participants		Age		Cough severity score^a^	
	Intervention	Comparator	Intervention	Comparator	Intervention	Comparator
Study	*n*	*n*	Mean	Mean	Mean	Mean
Ayazi et al. 2017	67	20	N/A	N/A	3.9	3.6
Canciani et al. 2014	51	51	4.9 (1.0)	4.4 (1.1)	2.6	2.6
Carnevali et al. 2021	54	52	4.4	4.4	2.1	2.1
Cohen et al. 2012	199	71	2.4	2.4	3.7	3.6
Cohen et al. 2017	78	72	3.5	3.6	3.8	3.5
Miceli Sopo et al. 2015	71	63	N/A	N/A	N/A	N/A
Nishimura et al. 2022	78	83	2.8	2.7	2.5	2.3
Paul et al. 2007	35	33	5.4	4.4	4.0	3.9
Shadkam et al. 2010	33	70/36^b^	N/A	N/A	3.8	3.9/3.9**
Waris et al. 2014	57	45/43^c^	N/A	N/A	3.0	2.8/2.9***

N/A = Not available

^a^The used Likert scales to assessed cough severity varied between 1–5 and 1–7 scaled versions

^b^Comparator groups were both over the counter medication and no treatment

^c^Comparator groups were placebo and salbutamol

### Risk-of-bias

**Fig. 2:**
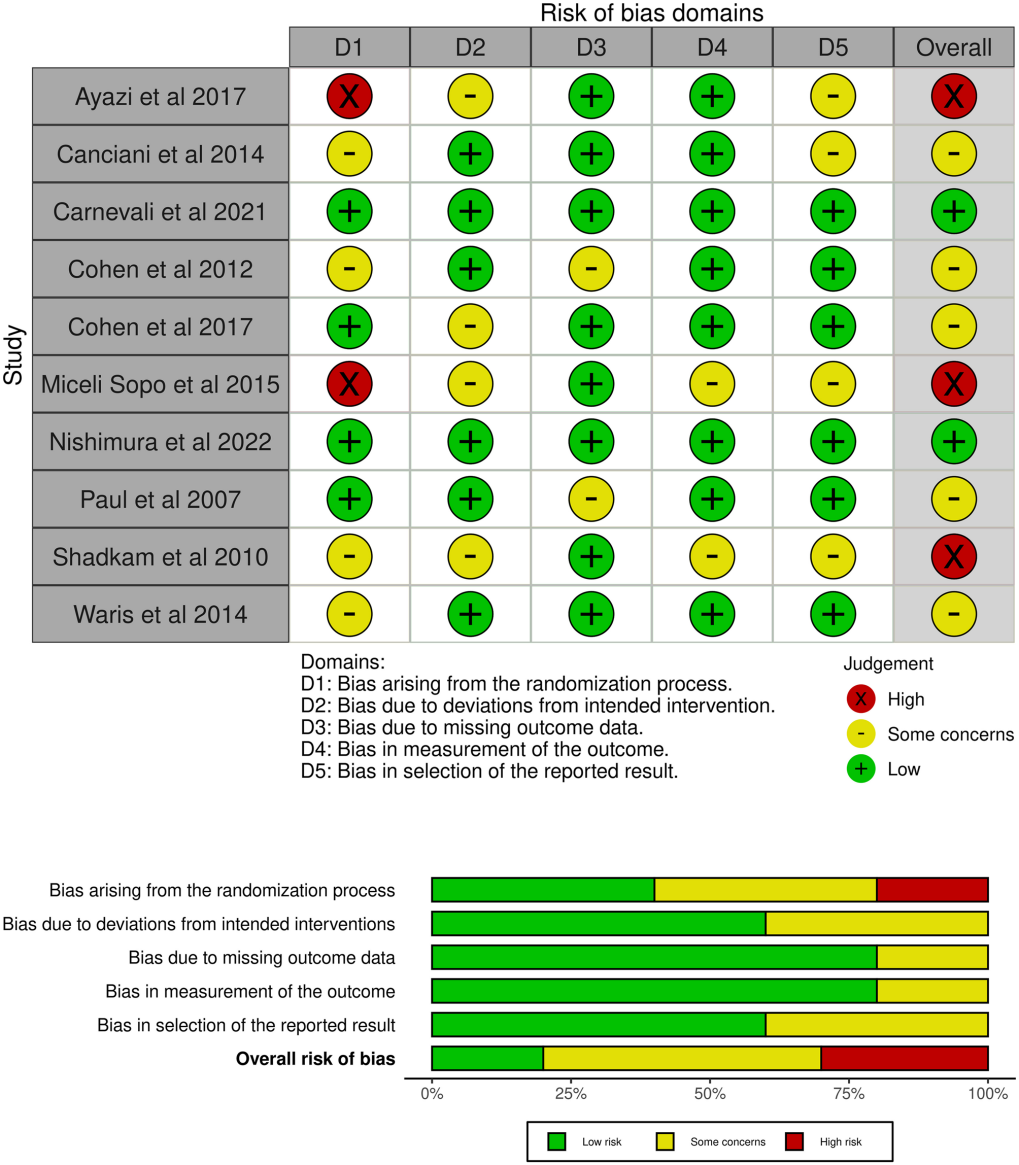
Risk of bias in the included studies assessed in the included studies in five domains and overall

Overall risk-of-bias was assessed to be high in three studies, with some concerns of risk-of-bias in six studies and a low concern in two studies (Fig. 2a). Most of the issues were detected in the bias arising from the randomization process and bias due to deviations from the intended interventions (Fig. 2b).

### Cough frequency

Ten studies measured cough frequency. Seven studies compared cough frequency between honey and placebo/no treatment and five studies compared cough frequency between honey and cough medication. The observed range of change from baseline favored treatment with honey over placebo/no treatment (0.0 to 1.1 points) and cough medication (0.2 to 0.9 points). The quality of the evidence was ranked as low (Table 2).

**Table 2:** Main findings of the included studies summarized by calculating the difference in change from baseline between the intervention and comparator groups. Overall effect presented as the range of the observed effect with the observed direction. Quality of the evidence presented according to GRADE methodology for outcomes

	Main outcomes			Secondary outcome
Study	Cough frequency	Cough severity	Child sleep	Harms/adverse events
***Honey vs placebo/no treatment***				
Canciani et al. 2014	+ 0.4 points	N/A	+ 0.5 points	None related to study products
Carnevali et al. 2021	+ 0.6 points	N/A	+ 0.8 points	None related to study products
Cohen et al. 2012	+ 0.8 points	+ 1.0 points	+ 0.7 points	2.0% in honey group, 1.4% in placebo
Nishimura et al. 2022	0.0 points	+ 0.1 points	0.0 points	5.1% in honey group, 1.2% in placebo group
Paul et al. 2007	+ 0.9 points	+ 0.5 points	+ 1.0 points	14.3% in honey group and 0% in placebo group
Shadkam et al. 2010	+ 1.1 points	+ 1.2 points	+ 1.1 points	N/A
Waris et al. 2014	+ 0.2 points	+ 0.3 points	+ 0.5 points	No difference stated by authors
**Total range of observed effects**	0.0 – + 1.1 points	+ 0.1 – + 1.2 points	0.0 – + 1.1 points	0.0%-14.3% in honey group and 0.0%-1.4% in placebo group
**Likely direction of the observed effect**	Favors honey	Favor honey	Favors honey	Favors comparator
**Evidence quality**	Low^a^	Low^a^	Low^a^	Very low^b^
***Honey vs cough medicine***				
Ayazi et al. 2017	+ 0.9 points	+ 1.3 points	+ 1.1 points	none related to study products
Cohen et al. 2017	+ 0.7 points	+ 0.7 points	+ 0.8 points	N/A
Miceli Sopo et al. 2015	Composite outcome (which included these three outcomes) had no difference			N/A
Paul et al. 2007	+ 0.5 points	+ 0.3 points	+ 0.6 points	14.3% in honey group, 3.0 in comparator group
Shadkam et al. 2010	+ 0.4 points	+ 0.5 points	+ 0.5 points	N/A
Waris et al. 2014	+ 0.2 points	+ 0.2 points	− 0.2 points	No difference stated by authors
**Total range of observed effects**	+ 0.2 – + 0.9 points	+ 0.2 – + 1.3 points	− 0.2 – + 1.1 points	N/A
**Likely direction of the observed effect**	Favors honey	Favors honey	Favors honey	N/A
**Evidence quality**	Low^a^	Low^a^	Low^a^	Very low^a^

^a^GRADE: Downgraded due to likely imprecision, indirectness, and risk of bias

^b^GRADE: Downgraded due to all modalities

### Cough severity

Seven studies measured cough severity. Five studies compared honey to placebo/no treatment, and the observed range of effect for the change from baseline was 0.1 to 1.2 points higher in the honey group. Five studies compared honey to cough medication, and the observed range of change from baseline was 0.2 to 1.3 points higher in the honey group. The quality of the evidence was ranked low in both comparisons (Table 2).

### Quality of a child’s sleep

Ten studies measured the quality of a child’s sleep. The observed range of effect, as measured by the change from baseline, was 0.0 to 1.1 points higher in the honey group in comparison to the placebo/no treatment group and −0.2 to 1.1 points higher in the honey group in comparison to the cough medication group (low quality evidence; Table 2).

### Adverse events

Seven studies measured adverse events. In the honey group, the reported percentage of children with an adverse effect ranged from 0.0 to 14.3%. Adverse events mostly included nausea and vomiting. In the placebo/no treatment group, the percentage of children with an adverse effect ranged from 0.0 to 1.4%; in the cough medication group, it ranged from 0.0 to 3.0%. Additionally, one study stated that there were no differences in the rate of adverse effects in honey or cough medicine groups, but it did not report specific results. The quality of the evidence was ranked as very low (Table 2).

## Discussion

Honey seems to be more effective than a placebo/no treatment or cough medications in relieving symptoms of acute cough in children. Overall, the quality of the evidence was low and meta-analysis was not possible due to heterogeneous reporting and the lack of key information in the included studies. Therefore, a combined statistical estimate could not be produced.

Our results are in line with previous meta-analyses. The Cochrane review from 2018 stated that honey could be effective in children in comparison to placebo/no treatment and most types of cough medication [11]. Compared to this previous review, we were able to include four additional studies. Of the additional studies, one double-blinded placebo-controlled study showed a clear null effect [23]. Although we did not perform meta-analysis, the systematic review of the range of observed effects indicated indirectly that honey would be more effective than placebo/no treatment and cough medication.

Interestingly, the most recent meta-analysis, which included adults, stated that honey could be an alternative to antibiotics, but we were unable to find a single study in which antibiotics would have been the comparator group [12]. The conclusion that can be made based on their analysis and the current report is that honey may be effective in reducing symptoms in children suffering from acute cough. Unfortunately, the quality of the reporting of adverse effects was low. It seems that honey may have a higher possibility of causing adverse events than placebo/no treatment. Based on our results, no sufficient comparison between honey and cough medication can be made. However, this is not a limitation as, generally, the use of cough medications should be avoided in children.

Most of the included studies reported the outcome as a change from the baseline assessed using a Likert scale (varying from 5 to 7 point scale). The observed differences in the change from baseline ranged between 0.0 and 1.2 points in these scales. Unfortunately, none of the included studies discussed the minimal important clinical difference [27]. Furthermore, all of the studies utilized a parent-reported outcome measurement; none of the studies aimed to analyze objective outcomes, such as cough frequency, measured by recording sounds while a child was sleeping. It is interesting to note that, in the included studies the patients in the placebo/no treatment group showed improvement, which indicates that usually a cough will spontaneously resolve in children.

Based on our results, it seems feasible to tell the parents that honey is a possible and likely effective treatment for acute cough but it may cause some adverse reactions (mainly vomiting or nausea). Future randomized studies with a double-blinded, placebo-controlled design are needed to determine the effectiveness of honey and the rate of adverse events before it is possible to make stronger recommendations for clinical practice.

### Protocol deviations

In comparison to the original protocol, we decided not to synthesize the results as a meta-analysis due to the high heterogeneity in the given interventions and comparator groups and due to lack of vital information for the pooling of the results, as nearly all of the included studies lacked SDs for the selected outcomes. Therefore, the results of such a pooled meta-analysis would be vague and have a high risk of bias. Instead, we decided to analyze the range of observed effects as doing so only requires evaluating the effect of estimate measures. However, this decision was made after publication of the protocol, which can be seen as a clear protocol deviation.

### Strengths and limitations

We decided to conduct our systematic review according to the original protocol regarding the study question and outcome assessment, which can be seen as a strength of this review. Furthermore, we did not forcefully conduct a meta-analysis based on input variables. However, a limitation of this review is that we deviated from the intended protocol by changing our statistical analysis plan. The main limitation to the interpretation of the results was the poor reporting on and coverage of adverse events. Due to limited reporting of uncertainty estimates in the original studies (missing standard deviations, interquartile ranges, CI, *p*-values), we were unable to provide uncertainty estimates for our range of observed effect estimate. This makes the results harder to interpret.

## Conclusion

This review presents low quality evidence that honey may be more effective than cough medication or placebo/no treatment in relieving symptoms and improving sleep in a child with acute cough. However, only two studies had low risk of bias, and one showed benefit while the other did not show evidence of benefit. Thus, further randomized, placebo-controlled, blinded trials are needed to confirm the efficacy and improve the quality of evidence regarding the use of honey to treat acute cough in children.

### Supplementary Information

Below is the link to the electronic supplementary material.

Supplementary file1 (DOCX 18 KB)

Supplementary file2 (DOCX 31 KB)

## Data Availability

All data extracted during the review process are available in the manuscript and supplement.
