# Supplementary material for: Honey for acute cough in children — a systematic review
Source: Eur J Pediatr. 2023 Jun 25;182(9):3949–56. doi: 10.1007/s00431-023-05066-1 (PMC10570220; doi:10.1007/s00431-023-05066-1)
Supplement: Supplementary file 1 — Supplementary file1 (DOCX 18 KB) [file 431_2023_5066_MOESM1_ESM.docx]

**Supplementary table 1** Background characteristics of the included studies.

| Study | Country | Study period | Study setting | Blinding | Cough duration | Age | Inclusion criteria | Exclusion criteria | Intervention | Comparator | Treatment duration | Main outcome measures | Funding | COI |
| --- | --- | --- | --- | --- | --- | --- | --- | --- | --- | --- | --- | --- | --- | --- |
| Ayazi et al 2017 | Iran | 2013-2014 | Children’s Hospital Outpatient clinic | Unblinded | <7 days | 1-12 years | presence of a viral URTI-induced cough in a previously healthy subject with no past medical history. The manifestations of viral URTIs included congestion, fever (an oral temperature of less than 39C), pharyngitis, malaise, or headaches. | pneumonia, laryngotracheobronchitis, sinusitis, asthma, allergic rhinitis, frequent hospitalization, recent administration of DPH to children, and use of medications that affected the sleep of the parents. Prior use of any cough or cold medication or honey | Honey | Diphenhydramine | two weeks | cough frequency, cough severity, bothersome nature of the cough, and the children's and parents' sleep quality. | None received | The authors had none |
| Canciani et al 2014 | Italy | Not presented | Pediatric referral unit | Double | 7 to 21 days | 3-6 years | Cough for seven days and age | Not specified | Grintuss syrup (contains honey) | placebo | 8 days | changes in the day- and night-time cough score | Not reported | One author had connection to study medicine company |
| Carnevali et al 2021 | Italy | 2019 | Pediatric referral unit | Double | 3 to 21 days | 3-6 years | Cough for three days and age | cough lasting more than 3 weeks, children with a history of obstructive pulmonary diseases, heart diseases, cystic fibrosis, diabetes, neurological diseases, and immunodeficiencies. | KalobaTUSS syrup (contains honey) | placebo | 8 days | change in the night-time and day-time cough score | None received | Four of the authors were employed by the company that produces the study medicine |
| Cohen et al 2012 | Israel | 2009 | Pediatric community clinic | Double | <7 days | 1-5 years | Cough duration and age | signs or symptoms of asthma, pneumonia, laryngotracheobronchitis, sinusitis, and/or allergic rhinitis. Patients were also excluded if they had used any cough or cold medication or honey on the night before entering the study. | Honey | placebo | one day | cough frequency. | Israel Ambulatory Pediatric Association, Maternal Infant Nutrition Research Institute, Honey Board of Israel. | The authors had none |
| Cohen et al 2017 | Israel | 2013-2014 | Pediatric community clinic | Single | <7 days | 2-5 years | Cough duration and age | signs or symptoms of asthma, pneumonia, chronic cough, stridor or laryngotracheobronchitis, sinusitis, chronic cardiac or pulmonary condition, allergic rhinitis, or if they had used steroid treatment, antihistamines or any cough or cold medication or honey for 24 hours before presentation. | Grintuss syrup (contains honey) | carbocysteine syrup | four days | cough frequency, cough severity, bothersome nature of the cough, and the children's and parents' sleep quality. | None received | The authors had none |
| Miceli Sopo et al 2015 | Italy | 2013 | Primary care pediatric facilities | Unblinded | <8 days | 1-14 years | Cough duration and age | asthma, pneumonia, streptococcal tonsillitis, sinusitis, bronchitis, allergicrhinitis; prior use of analgesic medications for cough over the counter products, oral antihistamines, cortisone, NSAIDs, or honey | Honey | dextromethorphan and levodropropizine | 3 days | frequency and bothersome nature of cough | None received | The authors had none |
| Nishimura et al 2022 | Japan | 2021-2022 | Pediatric community clinic | double | <8 days | 1-5 years | Cough duration and age | asthma, croup or any other obvious dyspnoea or wheezing. Prior cough medication. Antimicrobial treatment. COVID-19 positivity. | honey | placebo | 2 days | cough frequency, cough severity, bothersome nature of the cough, | None received | The authors had none |
| Paul et al 2007 | USA | 2005-2006 | General pediatric practice | Single | <8 days | 2-18 years | presence of rhinorrhea and cough for 7 or fewer days’ duration. Other symptoms may have included but were not limited to congestion, fever, sore throat, myalgias, and headache. | asthma, pneumonia, laryngotracheobronchitis, sinusitis, allergic rhinitis). They were also ineligible when they had a history of reactive airways disease, asthma, or chronic lung disease. Subjects were also excluded if on the prior use of medication. | Honey | no treatment or dextromethorphan | one day | Cough frequency, cough severity, bothersome nature of cough, and child and parent sleep quality. | National Honey Board, an industry-funded agency of the US Department of Agriculture. | One author had been a consultant to the Consumer Healthcare Products Association and McNeil Consumer Healthcare. |
| Shadkam et al 2010 | Iran | 2008-2009 | Not specified | Unblinded | >4 days | 2-5 years | rhinorrhea, sneeze, sore throat, and stuffed nose. Their coughing had lasted 5 days. | asthma, pneumonia, laryngotracheobronchitis, sinusitis, allergic rhinitis, chronic lung disease, congenital heart disease, malignancy, and diabetes. Prior use of cough medication. Parental use of sedative medication. | Honey | Dextromethorphan or Diphenhydramine or nothing | one day | cough frequency, cough severity, and sleep quality in children and their parents | University funded | The authors had none |
| Waris et al 2014 | Kenya | 2010-2012 | University hospital pediatric clinic | Double | Not specified, acute | 1-12 years | acute upper respiratory tract infection | prior use (48 hours) of any cough mixture, study agents, oral anti-histamines, nasal decongestants, steroids or anti-biotics. Atopy, asthma or any chronic lung disease. Hospitalization for lower respiratory tract infection in the past six months. | Honey | Placebo or salbutamol | five days | cough frequency, cough severity, and sleep quality in children and their parents | Not reported | Not reported |
